# Creation of Dimicleft radiological cleft phantom skulls using reversed virtual planning technique

**DOI:** 10.1259/dmfr.20230121

**Published:** 2023-06-22

**Authors:** Laura Pálvölgyi, Artúr Kesztyűs, Sohaib Shujaat, Reinhilde Jacobs, Krisztián Nagy

**Affiliations:** 1 Center for Facial Reconstruction, 1st Department of Paediatrics, Semmelweis University, Budapest, Hungary; 2 King Abdullah International Medical Research Center, Department of Maxillofacial Surgery and Diagnostic Sciences, College of Dentistry, King Saud bin Abdulaziz University for Health Sciences, Ministry of National Guard Health Affairs, Riyadh, Saudi Arabia; 3 OMFS IMPATH Research Group, Department of Imaging & Pathology, Faculty of Medicine, KU Leuven & Oral and Maxillofacial Surgery, University Hospitals Leuven, Leuven, Belgium; 4 Department of Dental Medicine, Karolinska Institutet, Stockholm, Sweden

**Keywords:** cone-beam computed tomography, diagnostic imaging, three-dimensional imaging, cleft palate, imaging phantoms

## Abstract

**Objectives:**

The aim of this technical report was to develop customized pediatric phantoms for cone-beam computed tomography (CBCT)-related research in cleft patients.

**Methods:**

Six human pediatric skulls (age: 5–10 years) were recruited. A cone-beam computed tomography (CBCT) scan was taken for each skull, followed by virtual modeling through the process of segmentation. An artificial cleft was designed and printed to be applied onto the skull for the creation of an artificial cleft. The skulls were covered with non-radiopaque tape and immersed in melted Mix-D soft tissue equivalent material. The resulting phantoms covered with Mix-D were assessed radiologically by two expert radiologists. These phantoms were referred to as Dimicleft pediatric skull phantoms.

**Results:**

Dimicleft phantoms were able to appropriately mimic *in vivo* circumstances. No gaps existed between Mix-D and bony tissue. Virtual planning allowed the optimal designing of an artificial cleft onto the phantom. The artificially created cleft was suitable to determine the size, location, and extent of the cleft.

**Conclusions:**

Dimicleft phantoms could act as a viable alternative to other commercially available options for assessing image quality and optimizing CBCT protocols in cleft patients for diagnostics and three-dimensional treatment planning.

## Introduction

Cleft lip and palate are one of the most common congenital craniofacial anomalies having both esthetic and functional implications. Cleft patients undergo numerous surgical interventions and require an adaptive treatment plan determined by a multidisciplinary team of oral surgeons, orthodontists, plastic surgeons, and speech therapists.^1,2^

Amongst various imaging techniques, cone-beam computed tomography (CBCT) has recently become a clinical standard for diagnostics and treatment planning in cleft patients, owing to its lower cost and reduced radiation exposure compared to conventional CT devices. Due to the continuous use of dentomaxillofacial CBCT imaging at different stages in cleft patients, it is indispensable to justify the radiation exposure and optimize scanning parameters.^3,4^ So far, various guidelines and positioning statements have been proposed for optimizing CBCT parameters, such as ALADA (as low as diagnostically acceptable) and ALADAIP (as low as diagnostically acceptable being indication-oriented) principles.^5–8^ In addition, an international project SEDENTEXCT was also developed to create an evidence-based guideline on the use of CBCT in dentistry. However, indication- and patient-specificity have not been addressed in cleft patients where similar acquisition parameters have been suggested for all patients irrespective of their age.

Owing to the availability of multiple CBCT devices with variable technical parameters, it is vital to create appropriate age- and indication-specific CBCT-based protocols, where personalized optimization studies should cover diagnostics, pre-surgical treatment planning, three-dimensional (3D) modeling, and 3D printing. Recently, a new European project from Euratom called ‘*Dentomaxillofacial pediatric Imaging: An Investigation Towards low dose Radiation-induced risks project'* (DIMITRA) also recommended to acquire images in cleft patients depending on the task at hand in order to reduce the radiation exposure.^9–13^ Thereby, optimization strategies are required which could considerably reduce the effective radiation dose in pediatric cleft patients without degrading image quality for the task at hand.^14,15^

These optimization protocols require a well-defined anatomic phantom, which should allow testing of both radiation dose and image quality simultaneously. For that purpose, several commercial anthropomorphic phantoms have been recommended that mimic hard and soft tissue and emit ionizing radiation similar to a real adult human. The main disadvantage of these phantoms is that they do not consider either age- or indication-specificity and rely on completely normal phantoms. Furthermore, they produce unsatisfactory CBCT scans for image quality assessment as the majority of them consist of water as the main constituent, whose volume is difficult to regulate around the phantom. This results in an unrealistic CBCT image.^16,17^ Although the soft tissue simulation material of these phantoms is capable of X-ray attenuation, the resulting images have increasing noise compared to *in vivo* CBCT images. Moreover, these phantoms are not designed to mimic human radiological anatomy accurately which further inhibits optimal evaluation of anatomical image quality unlike natural skull phantoms.^13^ To our knowledge, no pediatric age-specific phantoms are available with simulated cleft defects, which could allow a more realistic patient-specific approach toward optimizing CBCT acquisition parameters for reducing radiation exposure.

Therefore, the following technical report aimed to develop age-specific pediatric cleft phantoms specifically tailored for cleft patients which could allow for the optimization of CBCT scanning protocols and assess image quality for various diagnostic and treatment planning tasks.

## Methods and materials

### Natural skulls selection

Six pediatric skulls (age: 5–10 years) with well-preserved anatomical structures were obtained following Ethical approval from the Hungarian Natural Museum (SE RKEB number: 265a/2019, Budapest, Hungary). An initial CBCT scan of the skulls was acquired using NewTom VGi evo (NewTom, Verona, Italy) with a high-resolution protocol (Field of view: 12 × 8 cm, voxel size: 0.2 mm, New Tom VGI Evo, New Tom, Verona, Italy) for confirming their anthropological age and saved in Digital Imaging Communications in Medicine (DICOM) format for further processing. All skulls consisted of well-preserved bone and dental tissue without any large metallic restorations, dental posts, or implants. Dental germs (normal and malpositioned), missing teeth, and mesiodens were also detected.

### Segmentation, virtual design, and creation of artificial clefts

The CBCT DICOM files of skulls were imported individually into Mimics^®^ software (version 21, Materialise, Leuven, Belgium). Segmentation (volumetric reconstruction) of skeletal tissue was performed using predefined threshold values based on the image intensity. The region of interest (ROI) was cropped to the maxillary region for better visibility and less computing time. Fine adjustment of the segmented region was performed using region grow, multiple slice edit, and contour editing functions. Following segmentation, artificial clefts were designed on the skull’s left side as the frequency of cleft occurrence is higher on this side. Multiple-slice editing was applied to create a virtual cleft defect. This designing was performed by taking clinical and radiographic images of patients with cleft deformity as a reference.

**Figure 1.**
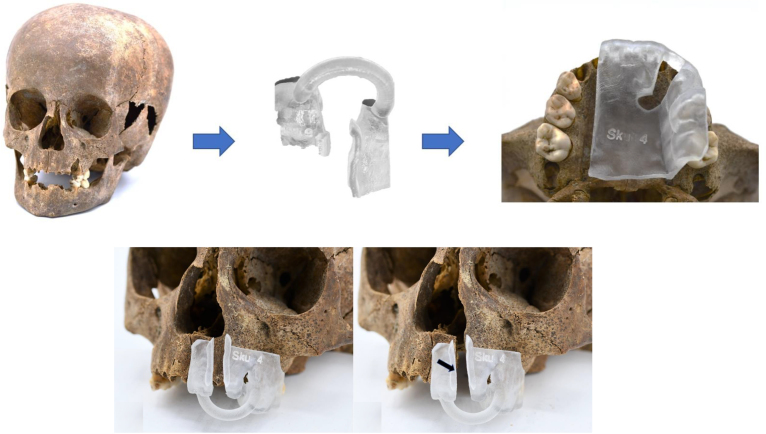
Creation of cleft following adaption of surgical template

Thereafter, both segmented skull and artificial cleft were saved in standard tessellation language (STL) format and imported to 3matic^®^ software (version 14, Materialise, Leuven, Belgium) for designing cutting guides. In order to mimic the pyramid-like shape of clefts, two cutting guides (frontal and palatal) were created. Firstly, two different surfaces were marked (frontal and palatal) on the segmented artificial cleft using the mark brush function. Thereafter, both guides were designed separately. For each guide “create a line” function was used in order to set two cutting angles. After applying the “extrusion”, “hollow”, and “boolean subtraction/wrap” operations, a base for the cutting guides was formed. For the frontal guide, a connecting ring and rim were made for more stable support of the instruments during cleft surgery. The cutting guides were fabricated with Objet30 Prime 3D printer (Stratasys, Rehotov, Israel) using Med610 resin (Stratasys, Rehotov, Israel). Thereafter, an experienced maxillofacial surgeon made artificial clefts onto the phantoms following the contours of the guides using piezoelectric and rotating surgical instruments (Figure 1).

### Skull coverage

**Figure 2.**
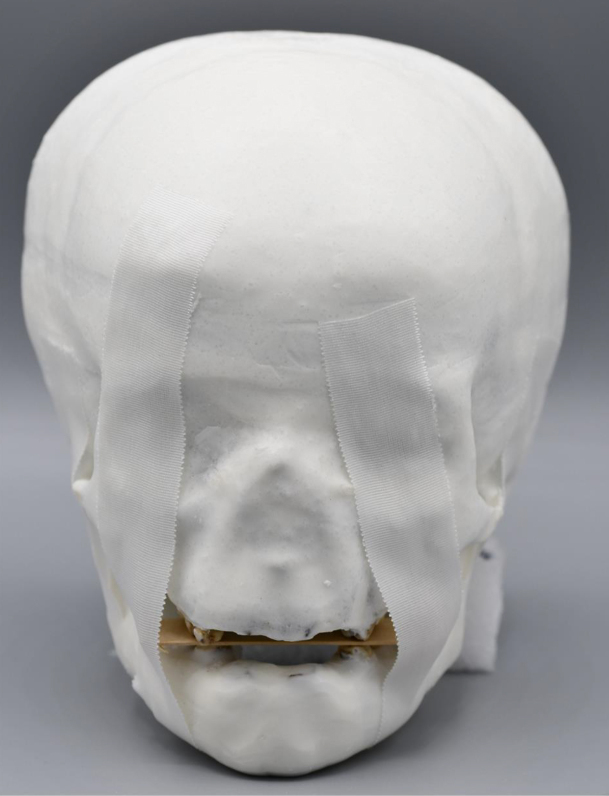
Coverage of skull with Mix-D

A soft tissue equivalent material called Mix-D was prepared and adapted onto the skulls with clefts as proposed by Brand et al^18^ (Figure 2), using the original recipe introduced by^19^ A 500 g of Mix-D was prepared in fractionated portions of 304 g of paraffin wax, 152 g of polyethylene, 32 g of magnesium oxide, and 12 g of titanium dioxide. Polyethylene was added after the preparation was completely mixed and melted, followed by another 20 min of heating. A fume hood was used for material preparation, manipulation, and skull coverage to assure chemical safety. Beforehand, the skulls were covered with crepe tape (paper with adhesive resin–rubber-based, 24 mm width; 3 M, Maplewood, MN) to avoid excessive infiltration of the material into the cranial cavities and interdental spaces. The skulls were immersed in melted solution until the superior orbital arch. When the Mix-D presented with a typical loss of gloss with pre-drying of the external surface, the skulls were re-immersed. This re-immersion process was repeated several times till a consistent and uniform layer of Mix-D surrounded the skull. After 24 h, the skulls were immersed in an inverted position by holding them at the region of zygomatic arches. Finishing and refinement were conducted with heated carving tools for removing excess material attached to the dental surfaces or interdental spaces. The final skull phantoms with clefts and Mix-D coverage were denoted as ‘Dimicleft pediatric skull phantoms’ and the whole collection was called “Dimicleft series”.^15^

### CBCT scanning

The quality of Dimicleft phantoms was assessed radiologically following the acquisition of CBCT images with a standardized, high-resolution protocol as previously applied for the initial scan. Two dentomaxillofacial radiologists assessed the image quality based on the presence of air bubbles, cracks or inhomogeneities, alignment of the covering material, and the appearance of created artificial clefts.

## Results

All the phantom skulls were able to appropriately mimic *in vivo* conditions with optimal image quality, which was confirmed by the radiologists. Some of the Mix-D infiltrated into the large bone cavities, such as the orbit, maxillary sinus, and nasal cavity. As this minimal infiltration was present superficially and was easily controlled and minimized with a non-radiopaque tape, it did not have any impact on either air density of sinus/nasal cavity or the image quality. Furthermore, no gaps existed between the soft tissue equivalent and bony tissue. Hence, confirms the similarity to a CBCT scan of a real patient, where both soft and hard tissue are perfectly aligned without any gaps.

The virtual planning allowed the optimal designing of an artificial cleft onto the phantom. In addition, 3D printing enabled the fabrication of templates that were ready to be transferred to the cleft site. The artificially created clefts in phantoms were suitable to determine the size, location, and extent of the cleft which is indispensable for planning surgical interventions. The complete virtual planning, template adaptation, and surgery were pretested on a 3D-printed Dimicleft phantom which also confirmed the fit and stability of the guides and the precision of the surgery.

## Discussion

The risk of radiation exposure in pediatric cleft patients has become a pressing issue since the risk of cellular damage is higher in such patients.^20^ The most common limitation of prior *in vivo* clinical studies is that the exposure aspects are ethically not well-tolerated and unacceptable, especially when pediatric patients are involved.^21^ Hence, it is necessary to devise pediatric age-specific phantoms for CBCT image quality assessment and optimization with different diagnostics and treatment planning protocols in cleft patients. So far, no such age- and indication-specific phantoms exist. Therefore, the following technical report focused on devising Dimicleft pediatric skull phantoms.

Dimicleft phantoms were able to reproduce realistic anatomical conditions, 3D visualization, and tactile feedback. Unlike unrealistic commercial phantoms, both two-dimensional (2D) and 3D image quality assessment and optimization strategies can be performed as these skulls are based on real pediatric patients. The proposed phantoms could be useful for devising low-dose protocols for adequately visualizing cleft and surrounding structures. This could further allow for a reduction in radiation dose when considering diagnostics, treatment planning, and outcome assessment in cleft patients. In addition, these phantoms could also help explore which CBCT protocol would be better for creating a 3D cleft model for surgical template designing and cleft bone grafting.

As far as translation of data from pediatric non-cleft phantoms to cleft patients is concerned, we believe image quality interpretation of a pristine non-cleft phantom without any defects would be different from that of a real cleft patient or natural cleft phantom possibly due to voxel gray values variability and impact of the pathological defect onto the surrounding structures quality. However, further *in vitro* research is recommended to confirm the image quality differences between normal and pathological indication-specific phantoms using different CBCT acquisition protocols before the findings can be translated to a real patient. Moreover, the proposed phantom could be useful for establishing a more personalized patient- and indication-specific standardization and optimization of different scanning parameters according to ALADA and ALADAIP principles, instead of relying on defect-free phantoms.^7^

The key parameters for both soft and hard tissue-mimicking materials in phantoms are tissue’s linear attenuation coefficient (LAC) for photoelectronic absorption, physical density, and effective atomic numbers.^22^ The main limitation of existing phantoms is that one of the mentioned materials' properties is not always present. Hence, Dimicleft phantoms could act as a viable alternative which is based on real pediatric phantom skulls and coverage with Mix-D has been well documented to optimally mimic soft tissue.

The Mix-D preparation and embedding technique were found to be feasible and reproducible. In addition, its ability to infiltrate the nasal cavities could be useful for mimicking pathological conditions such as the thickening of the sinus membrane, where infiltration can be regulated by protection with non-radiological tape. As the Mix-D coverage becomes dry, it gets difficult to insert any measurement tools into the surface which can lead to cracking. Therefore, it is not possible to construct slots for dosimeters. However, these skulls could be considered as most useful for setting optimal radiation doses in cleft patients besides assessing image quality.^22^ Moreover, Mix-D’s manual manipulation was not easily controllable and experienced staff are required for adapting it onto the skulls with sufficient thickness. As the layer-by-layer immersion process makes the surface brittle after solidification and is prone to cracking, hence, the surgical procedure was performed before the coverage. In the midst of these limitations, the following report was the first to provide a description of phantoms specifically tailored for pediatric cleft patients.

## Conclusion

Dimicleft phantoms could act as a viable and clinically oriented alternative for assessing image quality and optimizing CBCT protocols in pediatric cleft patients for tasks related to both diagnostics and 3D treatment planning, as it is able to yield clinically equivalent images. Future cleft-based radiological studies are recommended to be performed using such tailored phantoms for further improvement in radiological guidelines based on age- and indication-specificity.
